# Medical and economic benefits of telehealth in low- and middle-income countries: results of a study in four district hospitals in Mali

**DOI:** 10.1186/1472-6963-14-S1-S9

**Published:** 2014-05-12

**Authors:** Cheick Oumar Bagayoko, Diakaridia Traoré, Laurence Thevoz, Soumahila Diabaté, David Pecoul, Mahamoudane Niang, Georges Bediang, Seydou Tidiane Traoré, Abdrahamane Anne, Antoine Geissbuhler

**Affiliations:** 1 DER Santé Publique, Faculté de Médecine et d'Odonto-Stomatologie, Université de Bamako, Mali; 2 Centre d’Expertise et de Recherche en Télémédecine et E-santé, Hôpital Mère Enfant, Bamako, Mali; 3 Département de Radiologie et Informatique Médicale, Université de Genève, Suisse

**Keywords:** telehealth, EQUI-ResHuS project, LMICs, medical and economic benefits, district hospitals, télésanté, projet EQUI-ResHuS, PFR-PRI, avantages médicaux et économiques, hôpitaux de district

## Abstract

**Background:**

The aim of this study was to evaluate the impact of telehealth on 1) the diagnosis, and management in obstetrics and cardiology, 2) health care costs from patients’ perspectives, 3) attendance at health centres located in remote areas of Mali.

**Methods:**

The impact of telehealth on health care utilization, quality, and costs was assessed using a five-point Likert-scale based questionnaire consisting of three dimensions. It was completed by health care professionals in four district hospitals. The role of telehealth on attendance at health centres was also assessed based on data collected from the consultations logs before and during the project, between project sites and control sites. Referrals specific to the activities of the research study were also evaluated using a questionnaire to measure the real share of telehealth tools in increasing attendance at project sites. Finally, the cost savings achieved was estimated using the transport and lodging costs incurred if patients were to travel to the capital city for the same tests or care.

**Results:**

The telehealth activities contributed to improving medical diagnoses in cardiology and obstetrics (92.6%) and the patients’ management system on site (96.2%). The attendance records at health centres increased from 8 to 35% at all project sites during the study period. Patients from project sites saved an average of 12380 XOF (*CFA Francs*) or 25 USD (American dollar) and a maximum of 35000 XOF or 70 USD compared to patients from neighbouring sites, who must go to the capital city to receive the same care.

**Conclusion:**

We conclude that in Mali, enhanced training in ultrasound / electrocardiography and the introduction of telehealth have improved the health system in remote areas and resulted in high levels of appropriate diagnosis and patient management in the areas of obstetrics and cardiology. Telehealth can also significantly reduce the cost to the patient.

## Abstract (fr)

La présente étude visait à évaluer l’impact de la télésanté sur 1) le diagnostic et la gestion en obstétrique et en cardiologie, 2) les coûts des soins de santé du point de vue des patients, 3) l’achalandage des centres de santé situés dans les régions éloignées du Mali.

**Méthodes**

L’incidence de la télésanté sur l’utilisation, la qualité et les coûts des soins de santé a été évaluée au moyen d’une échelle de Likert comportant cinq points et composée de trois dimensions. Elle a été remplie par des professionnels de la santé de quatre hôpitaux de district. L’incidence de la télésanté sur l’achalandage des centres de santé a également été évaluée en fonction des données recueillies dans les cahiers de consultation avant et pendant le projet entre les sites du projet et les sites de contrôle. Les demandes de consultation en lien avec les activités de l’étude ont également été évaluées à l’aide d’un questionnaire afin de mesurer l’incidence réelle des outils de télésanté sur l’achalandage des sites du projet. Finalement, les économies réalisées par les patients ont été estimées en tenant compte des coûts de transport et de logement qu’ils auraient assumés s’ils avaient dû se déplacer dans la capitale pour être soumis aux mêmes tests ou recevoir les mêmes soins.

**Résultats**

Les activités de télésanté ont contribué à améliorer les diagnostics médicaux en cardiologie et en obstétrique dans une proportion de 92,6 % et le système de gestion sur place du patient dans 96,2 % des cas. Durant l’étude, l’achalandage consigné des centres de santé est passé de 8 % à 35 % à tous les sites couverts par le projet. Les patients des sites du projet ont économisé en moyenne 12 380 francs CFA ou 25 $US, et un maximum de 35 000 francs CFA ou 70 $US, comparativement aux patients des établissements voisins qui ont dû se rendre dans la capitale pour obtenir les mêmes soins.

Nous en venons à la conclusion qu’au Mali une meilleure formation en échographie ou en électrocardiographie et l’introduction de la télésanté ont bonifié le système de santé des régions éloignées en améliorant le niveau de diagnostics appropriés ainsi que la gestion des patients en obstétriques et en cardiologie. La télésanté peut également réduire de façon importante les coûts pour les patients.

## Background

The results of several pilot studies point to the role of Information and Communication Technologies (ICTs) or e-health as a key element in creating equitable health care systems and services in low- and middle-income countries (LMICs) [1-3].

The World Health Organization has recognized the important role of e-health in creating a suitable health care system in LMICs through the adoption of several resolutions by its member states: the first in May 2005 in Geneva, the second in Malabo in 2010, and recently another in May 2013 [4-6]. According to WHO, these resolutions promote e-health as a means of strengthening health systems, through different mechanisms, namely:

• Promote national policy, and both involvement and awareness in e-health, including the designation et use of champions for this purpose;

• Strengthen leadership and coordination for e-health, exploring the possibility of setting up multidisciplinary and intersectoral support mechanisms;

• Systematically build human capacity for e-health by introducing the teaching of ICTs in health training institutions;

• Make the necessary investments in infrastructure and e-health services;

• Endeavor to reach communities, including vulnerable groups, with appropriate services to meet their needs.

These recommendations are particularly relevant in a country such as Mali, with a large territory and an acute deficit of health care professionals, as well as an inequitable distribution of health professionals within the country. There is a large disparity between the capital city, Bamako, and the others administrative areas. According to the 2010 report on human resources by the Malian Department of Health, only 40% of physicians, 49% of nurses, and 59% of midwives work outside the capital city, and 80% of medical specialists are concentrated in Bamako [7].

The *EQUI-ResHuS: ICTs for Equitable Access to Health Human Resources* research project, which is qualified, motivated, and well-supported in Francophone Africa, was started in 2009 in Mali through funding from the Global Health Research Initiative (GHRI) under the *Africa Health Systems Initiative – Support to African Research Partnerships* competition [8]. The main research question was how Information and Communication Technologies could contribute to a better distribution of human resources in health [9]. To answer this question, a study was designed to evaluate the role of improved diagnostic imaging capacity and telehealth (which can be defined as the practice of distance medicine, using ICTs) in two rare specialties in Mali, namely: obstetrical imaging and cardiology. These are two priority areas because, outside Bamako, there is only one radiologist and no cardiologist in a country of 1,241,000 square kilometres. This fact often leads to necessary but costly medical referral and the patient's reticence to go to treatment in the capital, due to lack of funds.

The main activities at the district hospitals were the task shifting in obstetric ultrasound and cardiology, and continuing medical distance education. These were implemented using the tools developed within the RAFT network, a large telemedicine and eLearning network deployed throughout francophone Africa [10,11].

This work focused on two aspects of the evaluation: the impact on the short term health outcomes (diagnosis and treatment) and the economic impact from the patient’s perspective, measured using purposefully designed assessment tools. Specifically, the aim of our study was to assess the short-term medical and economic impact of telehealth in four district hospitals in Mali which participated in the project.

This study is important since there are few in the literature that clearly demonstrate the impact of ICTs in the health sector. Some have highlighted the effects of ICTs on morbidity and mortality [12], but studies related to the impact of telehealth in LMICs are rare [1,3].

## Methods

Our study was conducted in district hospitals in Bankass, Dioila, Kolokani, and Djenne, in rural Mali. Prior to the implementation of this study, cardiology and medical imaging had been delegated to general physician and midwives.

For the shifting of these tasks in ultrasound imaging and cardiology, a three week training of health care professionals was held in Bamako in order to develop basic technical skills. The goal of the EQUI-ResHuS project was not to train specialists in these areas but to teach district hospital medical staff how to conduct a good electrocardiogram (EKG) and ultrasound examination, and seek experts’ opinions on diagnoses and provision of care for patients remotely via the tools of telehealth. Thus, rural health centres were equipped with portable ultrasound imaging devices and EKGs, laptops and low-bandwidth internet connections, mostly using 3G USB-keys.

For continuing distance education, we used the RAFT’s Dudal platform, an interactive distance learning platform suitable for low-bandwidth internet connections [13].

Between March 2012 and March 2013, study participants presenting to the one of the four district hospitals with an obstetrical or cardiac problem were invited to participate and were enrolled prospectively to the study. All participants signed an informed consent form. The study was approved by the ethics committee of the Malian Computer Network and Medical Communication (*Réseau Informatique Malien d'Information et de Communication Médicale*, *REIMICOM*). This network includes several public and private health care institutions in Mali.

Three types of questionnaires were used to measure the impact of diagnostic imaging and telehealth on health care structures and on patients: one was designed for the medical evaluation, the second for economic activity, and the third one dealt with the impact on district hospitals’ activities.

Regarding medical evaluation, three key indicators were measured using a five level Likert scale (ranging from “completely agree” to “do not agree at all”). These indicators are related to the usefulness of tests for the diagnosis, the role of remote expertise in diagnosis, and the impact on patient management. The sample consisted of 215 participants for the first indicator, 103 for the second, and 211 for the last. The medical evaluation questionnaires were completed by physicians after the consultation with the patient.

To study the economic benefits, we compared the costs to patients in two scenarios: performing the diagnostic test locally versus performing it in Bamako, where patients are forced to travel when tests are not available in the local facility. Cost variables relating to transportation, doctor visits, medical exams, and cost of living were compared. The transportation and consultation costs are fixed and known in all localities. The costs of stays were obtained on the basis of interviews with patients. The questionnaires were filled by the project evaluation team during the retrospective patient interviews.

The impact of the project on the health centre was evaluated using one indicator: the effect of the project on the number of consultations, or attendance. To do so, the number of medical consultations in the health centres before the beginning of our study, in 2009, and during the study, in 2012, was estimated using a case-control pre-post study design, comparing data from each study centre to a geographically and demographically similar centre before and after the introduction of telehealth. In addition, a third questionnaire was sent to each site to determine the number of consultations and referrals that directly resulted from the project activities. A case-control study was used to evaluate attendance at health centres, in order to control for the general trend over the study duration, and avoid crediting the increase in attendance solely to the intervention. The control sites were: Koro for Bankass, Baraouéli for Dioila, Banamba for Kolokani, and Youwarou for Djenne.

EpiData Software version 1.5.0.10 (The EpiData Association, Odense, Denmark) was used to analyse the data from the questionnaires.

## Results

### The impact on care

The first variable, usefulness of ultrasound or EKG to establish diagnosis, was not helpful in only one case and a little helpful in 15 cases. Ultrasound and EKG were found to be helpful in 199 cases out of 215 which represent 92.6% of cases.

The second variable measured whether the initial diagnosis made by the physician has been changed after the tests or answer from the expert. We found that the diagnosis was little changed in only six cases, and not changed at all in 26 cases. In 73 cases out of 103, it was changed completely, a lot, or moderately.

The third variable measured whether the tests or input from the remote expert had modified the treatment. We found that treatment did not change at all in only 3 cases of 211 and changed a little bit in 5. Treatment was changed moderately or completely in 203 of 211 cases (96.2% of cases). Table 1 summarizes the data from the medical evaluation.

**Table 1:** Distribution of the usefulness of the examination, conformity of diagnosis, and on-site support

		Bankass		Dioila		Djenne		Kolokani		Total	
	
Variables		N	Col %	N	Col %	N	Col %	N	Col %	N	Col %
Useful tests for diagnosis	Completely Very	16 12	50 37.5	3 39	5 65	1 72	1 72.7	19 5	79.2 20.8	39 128	18.14 59.53
	Average Slightly	4 0	12.5 0	14 4	23.3 6.7	14 11	14.1 11.1	0 0	0 0	32 15	14.88 6.98
	Not at all	0	0	0	0	1	1	0	0	1	0.47
	
	TOTAL	32	100	60	100	99	100	24	100	215	100.00

Changes applied to diagnosis by expert	Completely Very	0 0	0 0	1 1	3.8 3.8	2 39	2.7 52.7	0 0	0 0	3 40	2.91 38.83
	Average Slightly	0 0	0 0	9 2	34.6 7.7	21 4	28.4 5.4	0 0	0 0	30 6	29.13 5.83
	Not at all	0	0	13	50	8	10.8	3	100	24	23.30
	
	TOTAL	0	0	26	100	74	100	3	100	103	100.00

Changes in patient care based on remote expert’s recommendations	Completely Very	20 7	71.4 25	25 33	41.7 55	10 51	10.1 51.5	22 2	91.7 8.3	77 93	36.49 44.08
	Average Slightly	1 0	3.6 0	2 0	3.3 0	30 5	30.3 5.1	0 0	0 0	33 5	15.64 2.37
	Not at all	0	0	0	0	3	3	0	0	3	1.42
	
	TOTAL	28	100	60	100	99	100	24	100	211	100.00

Taken together, these results demonstrate that telehealth tools deployed in district hospitals have a major impact on the diagnosis and treatment of patients.

### Impact on patients’ health expenditures

There are significant differences in patient health-related expenditures under the two diagnostic imaging scenarios (local vs. regional), in a country where wages are considerably low.

Patients at a project site saved an average of 12380 XOF (25 USD) and a maximum of 35000 XOF (70 USD), in a country with a per capita Gross Domestic Product (GDP) of 1300 USD per year.

The average and maximum costs of main health expenditure at a project site compared to the capital, Bamako, is summarized in Table 2. The differences are in favour of the project sites.

**Table 2:** Comparison of health expenditure between EQUI-ResHuS sites and Bamako

Variables related to health expenditure		Cost (XOF) Project Site	Cost (XOF) Bamako	Difference for project sites
Transportation	Average (SD) Min Max	215 (939.31) 0 3000	8585 (6095.32) 0 24000	8370 0 21000

Consultation	Average (SD) Min Max	1120 (431.03) 0 2000	1355 (816.54) 0 5000	235 0 3000

Tests	Average (SD) Min Max	4900 (1887.94) 0 8000	7800 (2809.66) 0 15000	2900 0 7000

Living	Average (SD) Min Max	770 (609.34) 0 6000	1645 (1662.29) 0 10000	875 0 4000

Total of average cost		7005	19385	***12380***

Total of minimum cost		0	0	0

Total of maximum cost		19000	54000	**35000**

### The impact on attendance in health centres

Compared to the control sites, there was an increase in the number of consultations in the study districts, except in Dioila and its control site, Baraouéli, where the number had decreased for both. The observed decrease in consultations in Dioila and Baraouéli was attributed to the fact that new health centres were created in these two districts, which are close to the district hospital itself, thus redirecting some patients to these new centres.

Although there was a general trend toward increased attendance at both project and related sites, it should be noted that the increase was more important in the project sites. The overall increase in the rate of attendance at EQUI-ResHuS sites was 79.8% while it was 44.9% in the control sites.

**Figure 1:**
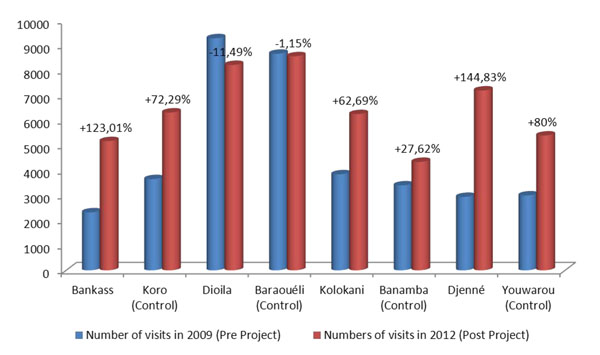
Change in the number of visits between intervention sites and control sites from 2009-2012

The share of telehealth in the increased attendance was measured at the project sites. Based on our findings, telehealth tools accounted for an increase in attendance at the project centres: 35% in Dioila District Hospital, 10% in Bankass, and 8% in Kolokani and Djenne (Figure 1). The appeal of telehealth is mainly reflected by an increase in patient referrals from other district hospitals to project district hospitals.

It is important to note that before the project, no district hospital had referred patients to the study sites.

## Discussion

The results of our study show that telehealth has a positive influence on the improvement of the medical decision making in remote area, on the reduction of health care costs for patients, and on increasing attendance and appeal of the health centres.

Our study was conducted on four EQUI-ResHuS project pilot sites and four control districts to determine the influence of telehealth on attendance. One of the limitations of our study was the lack of usable statistics about consultations for the previous years, before the start of the project. This limitation did not affect our results because we were able to determine the proportion of telehealth tools in increasing attendance at health centres. To compare data on attendance before and after the project, we used 2009 data for the initial situation before implementation, and 2012 data after the full implementation of the project.

Regarding the medical study, the difference in participant numbers for the three questions is related to fact that all questions were not answered for all questionnaires. For example, the exam could be considered useful for a patient but this doesn't necessarily imply a case submission to an expert for a second opinion or recommendation. We can deduce that the district health professionals have been well-trained over time and did not need the opinion of an expert for all cases. This fact is confirmed by the responses to the second question of the medical evaluation questionnaire (change applied to diagnosis by expert) where there is a good diagnosis concordance between the district health professionals and experts.

In the economic analysis that we conducted, the minimum cost of all variables is zero, because there is a system to provide free medical services in Mali for specific cases. The criteria for this free benefit may be different from one structure to another (local employees, patients in financial need, etc.).

Regarding the results of our study, the literature seems poor in terms of real impact of telehealth on health systems in LMICs. There is, however, much more evidence of the impact of health information systems in developed countries. For example, Amarasingham et al. [12] demonstrate that clinical information systems in hospitals can reduce morbidity, mortality, and costs.

Findings similar to our study are reported in India by Chandrasekhar and Ghosh [14] who conclude that ICTs can improve health in developing countries in many ways: continuing medical education, improving service delivery, possible reduction of costs, amongst others. Our study was able to demonstrate these aspects with quantitative results.

Elsewhere, the role of telemedicine in the organization of care and its requirements for successful implementation were described [15], but its actual impact has not yet been satisfactorily studied in LMICs such as Mali, hence the merit of this study despite some shortcomings [16].

Our results highlight the short-term medical and economic impact that telehealth can have in remote areas and it should serve as a case study for developing countries, especially in sub-Saharan Africa.

## Conclusions

In light of our study, we can affirm that telehealth has a positive impact on the quality of care, health care costs reduction, and increasing the appeal of participating district hospitals. However, a long-term impact study is necessary to demonstrate other long-term impacts such as the effect on human resources and health outcomes. In light of these results, telehealth can be a tool to improve equity in access to health in LMICs. Indeed health care becomes accessible to the most marginalized, namely those living in remote areas.

We conclude with this quote from a patient with heart disease that we interviewed during field visits: "the mere fact of asking me to go to Bamako (capital of Mali) for a test worsens considerably my illness. Therefore, for me this project is a miracle".

## List of abbreviations

EKG: Electrocardiogram; EQUI-ResHuS: Technologies de l'Information et de la Communication pour un Accès Equitable aux Ressources Humaines en Santé; GDP: Gross Domestic Product; GHRI: Global Health Research Initiative; ICTs: Information and Communication Technologies; LMICs: Low- and middle-income countries; RAFT: Réseau en Afrique Francophone pour la Télémédecine; REIMICOM: Réseau Informatique Malien d'Information et de Communication Médicale; USD: United States dollars; WHO: World Health Organization; XOF: West African CFA (Communauté Financière d'Afrique) franc

## Competing interests

The authors declare that they have no competing interests.

## Authors' contributions

COB and AG designed the research project and conducted the research. COB wrote the first draft of the manuscript. COB, AA and AG conducted the literature review.

LT, SD, and DP designed the first version of the questionnaire. AG, CO, and DT reviewed the questionnaire. SD, MN, and STT collected data. DT and GB carried out the statistical analysis. COB wrote the final version of the paper. COB, DT, and AG reviewed the final version.

## Declaration

The publication costs associated with this article are funded by Foreign Affairs, Trade and Development Canada and the International Development Research Centre through the Global Health Research Initiative.

This article has been published as part of *BMC Health Services Research* Volume 14 Supplement 1, 2014: Uptake and impact of research for evidence-based practice: lessons from the Africa Health Systems Initiative's research component. The full contents of the supplement are available online at http://www.biomedcentral.com/bmchealthservres/supplements/14/S1
